# Multi-Omics Analysis to Understand the Effects of Dietary Proanthocyanidins on Antioxidant Capacity, Muscle Nutrients, Lipid Metabolism, and Intestinal Microbiota in *Cyprinus carpio*

**DOI:** 10.3390/antiox12122095

**Published:** 2023-12-10

**Authors:** Rui Jia, Yiran Hou, Wenrong Feng, Munkhjargal Nomingerel, Bing Li, Jian Zhu

**Affiliations:** 1 Key Laboratory of Integrated Rice-Fish Farming Ecology, Ministry of Agriculture and Rural Affairs, Freshwater Fisheries Research Center, Chinese Academy of Fishery Sciences, Wuxi 214081, China; jiar@ffrc.cn (R.J.); houyr@ffrc.cn (Y.H.); fengwenrong@ffrc.cn (W.F.); 2 Wuxi Fisheries College, Nanjing Agricultural University, Wuxi 214081, China; nomibgerel999@gmail.com

**Keywords:** proanthocyanidins, intestinal microbiota, multi-omics, muscle nutrients, PPAR signaling pathway, *Cyprinus carpio*

## Abstract

Proanthocyanidins (Pros), a natural polyphenolic compound found in grape seed and other plants, have received significant attention as additives in animal feed. However, the specific mechanism by which Pros affect fish health remains unclear. Therefore, the aim of this study was to investigate the potential effects of dietary Pro on common carp by evaluating biochemical parameters and multi-omics analysis. The results showed that Pro supplementation improved antioxidant capacity and the contents of polyunsaturated fatty acids (n-3 and n-6) and several bioactive compounds. Transcriptomic analysis demonstrated that dietary Pro caused an upregulation of the sphingolipid catabolic process and the lysosome pathway, while simultaneously downregulating intestinal cholesterol absorption and the PPAR signaling pathway in the intestines. Compared to the normal control (NC) group, the Pro group exhibited higher diversity in intestinal microbiota and an increased relative abundance of *Cetobacterium* and *Pirellula*. Furthermore, the Pro group had a lower Firmicutes/Bacteroidetes ratio and a decreased relative abundance of potentially pathogenic bacteria. Collectively, dietary Pro improved antioxidant ability, muscle nutrients, and the diversity and composition of intestinal microbiota. The regulation of lipid metabolism and improvement in muscle nutrients were linked with changes in the intestinal microbiota.

## 1. Introduction

Aquaculture plays a vital role in supplying high-quality protein and essential micronutrients for human consumption, contributing to human health and overall well-being. However, in intensive aquaculture production, several factors, such as high stocking density, excessive feeding, and fluctuating conditions, have made it more susceptible to disease outbreaks [1]. To mitigate economic losses, various veterinary drugs, especially antibiotics and chemical agents, are extensively utilized in aquaculture for disease prevention and treatment [2]. Despite their effectiveness, the use of veterinary drugs is increasingly limited due to their adverse effects on the environment and human health [3]. As consumers are becoming more concerned about organic and environmentally friendly food, the utilization of natural products such as plant extracts or probiotics in aquaculture has been proposed as a possible solution [4,5]. In recent years, medicinal plants and their extracts have received considerable attention as eco-friendly and efficient alternatives to chemical agents [6]. They have been found to offer various beneficial effects to aquatic animals, such as stress reduction, growth promotion, appetite stimulation, and immunity improvement [7]. These effects are attributed to the presence of active compounds such as polysaccharides, alkaloids, tannins, saponins, flavonoids, or essential oils [6,8]. Furthermore, medicinal plants and their derivatives are commonly used as diet additives in aquaculture due to their ease of preparation, low cost, and minimal adverse effects on both the fish and the environment [4,9].

Proanthocyanidins (Pros), also known as condensed tannins, are a type of natural polyphenolic compound distributed in various plant parts, including stems, leaves, flowers, seeds, and fruit [10]. They are polymerized by flavan-3-ol subunits (i.e., catechin and epicatechin) and produced by the flavonoid biosynthetic pathway [11]. In recent years, there has been a growing interest in the research into Pros due to their attractive nutritional properties and perceived health benefits. Pros possess a variety of biological activities, such as antioxidative, free radical scavenging, anti-inflammatory, immune-stimulating, anti-viral, and cardio-protective features [12]. An in vitro study showed that Pros have the strongest antioxidant activity among more than 100 types of representative phenolic compounds [13]. They have also been found to improve intestinal permeability and increase microbial diversity in response to diet-induced unfavorable changes in the intestine [14,15]. Moreover, Pros can alleviate cardiovascular diseases [16], ameliorate the pesticide rotenone-induced mitochondrial respiration anomalies [17], and inhibit inflammation by modulating the NF-κB pathway [18]. Therefore, it is recommended to moderately enhance the intake of Pro-enriched food, as this may contribute to the prevention of chronic diseases and improve health conditions in both humans and animals [19].

In aquaculture, Pros have been investigated as dietary supplements, and their positive effects on various fish species have been demonstrated. Dietary Pro was reported to promote growth and improve serum biochemistry parameters related to health status in tilapia (*Oreochromis niloticus*) [20]. Wang et al. [21] showed that the weight gain, feed utilization, and growth performance of juvenile American eel (*Anguilla rostrata*) were increased when Pros were incorporated into their diet. In another study, Pros alleviated cadmium toxicity in pearl gentian grouper (*Epinephelus fuscoguttatus* female *× Epinephelus lanceolatus* male) via enhancing antioxidant ability [22]. Furthermore, Pros have been demonstrated to attenuate growth retardation and low immunoglobulin level induced by histamine in American eels [23], as well as mitigate hepatic lipid accumulation and inflammation caused by a high-fat diet in grass carp (*Ctenopharyngodon idella*) [24]. It is apparent that previous studies in aquatic animals have primarily focused on investigating the impact of Pros on growth and biochemical parameters. However, there is a scarcity of data regarding alterations in muscle nutrients, lipid metabolism, and intestinal microbiota in cultured fish after Pros feeding, particularly the lack of multi-omics analysis data to elucidate the underlying mechanism of Pros’ actions.

With the rapid advancement of molecular biology and bioinformatics, multi-omics methodologies are gaining recognition as powerful tools for understanding the biological processes of aquatic organisms and their interactions with environmental factors. These methodologies, including genomics, transcriptomics, proteomics, metabolomics, and microbiomics, can provide comprehensive insights into the intricate workings of biology systems. In response to environmental stressors, multi-omics analysis has revealed new pathways in fish that could be pivotal in understanding how these organisms adapt and survive [25,26]. Moreover, multi-omics analyses have facilitated the elucidation of crucial mechanisms involved in fish diseases, contributing to the treatment options and comprehension of pathogenic processes [27]. For example, Li et al. employed both transcriptomic and proteomic analyses to elucidate the beneficial impact of resveratrol on lipid metabolism disorders induced by a high-fat diet in red tilapia [28]. Extensive research evidence suggests that multi-omics approaches are capable of revealing a wider array of mechanisms in the fields of toxicology, pharmacology, physiology, and pathology of fish.

Common carp (*Cyprinus carpio*), a globally distributed and consumed species, is commonly used as an experimental animal in various research fields including pharmacology, toxicology, pathology, and nutrition. In aquaculture, it is frequently utilized for screening medicinal plant extracts and assessing their pharmacological effects [29,30]. Therefore, in this study, we selected common carp as the experimental animal to evaluate the impact of Pros on muscle nutrients, lipid metabolism, and intestinal microbiota via multi-omics analysis. For this purpose, the fish were fed diets supplemented with different doses of Pro (0, 0.2, 0.4, and 0.8 g/kg diet) for 10 weeks, and the effects on growth performance, antioxidant status, muscle metabolites, and intestinal transcriptome profiling and microbiota were investigated. To the best of our knowledge, this is the first report to evaluate the beneficial effects of Pro on cultured fish using multi-omics analysis.

## 2. Materials and Methods

### 2.1. Experimental Design and Sampling

The experimental common carp (average weight 41.6 ± 2.15 g) were obtained from the farm of Freshwater Fisheries Research Center (FFRC, Wuxi, China). Healthy fish were selected and kept in a recirculating water system for 7 days to adapt to the experimental conditions. After domestication, the carp were randomly divided into 4 groups and fed diets containing different amounts of Pro (0, 0.2, 0.4, and 0.8 g/kg) for 10 weeks (Figure S1). Each group consisted of 30 fish, distributed evenly across 3 tanks (10 fish/tank). The 0 g/kg Pro group served as the normal control (NC). The fish were fed experimental diets (Table S1) twice a day (8:30 a.m. and 4:00 p.m.) to satiation. The Pro from grape seed (CAS 4852-22-6; purity, 95%) used in this study was purchased from Absin Biotechnology Co., Ltd (Shanghai, China).

After 10 weeks of farming, all fish were weighed under anesthesia using MS-222 (100 mg/L, Sigma, St. Louis, MO, USA). Subsequently, blood, liver, gills, muscle, and intestines were collected for the measurement of antioxidant parameters. From each group, 9 fish (3 fish/tank) were randomly selected for sampling. All collected samples were stored at −80 °C until further use. For analysis of metabolome, transcriptome, and 16S rRNA sequencing, as well as amino acid and fatty acid composition, muscle and intestinal tissues were collected from 12 randomly selected fish (4 fish/tank) in the NC and 0.8 g/kg Pro groups. These tissues obtained from three individuals were pooled together to form one sample [31,32]. The samples were flash-frozen in liquid nitrogen and sent with dry ice to Gene Denovo (Guangzhou, China) for sequencing. The experiment was conducted with consideration for animal welfare, and the animals used in this study were approved by the FFRC. During the experiment, one fish died in each of the NC, 0.2 g/kg Pro, and 0.8 g/kg Pro groups, while no fish died in the 0.4 g/kg Pro group.

### 2.2. Measurement of Antioxidant Parameters

The blood was centrifuged at 5000 r/min for 10 min at 4 °C to obtain the serum. The liver, gill, muscle, and intestine tissues were homogenized with 9 times (*w*/*v*) normal saline at 4 °C. In serum, liver, gill, muscle, and intestine tissues, 9 samples from each group were used to detect the antioxidant parameters, including malondialdehyde (MDA), superoxide dismutase (SOD), glutathione peroxidase (Gpx), total antioxidant capacity (T-AOC), and glutathione (GSH). Kits for measuring T-AOC, SOD, and GSH were supplied by Nanjing Jiancheng Bioengineering Institute (Nanjing, China). The Gpx kit was provided from Suzhou Grace Biotechnology Co., Ltd. (Suzhou, China), while the MDA kit was ordered from the Beyotime Institute of Biotechnology (Nantong, China). SOD activity was measured at an absorbance of 450 nm utilizing the WST-1 method, with calculation based on the production of WST-1 formazan. The T-AOC level was assessed using the FRAP assay at 593 nm, which quantifies the reduction of the Fe^3+^ to the Fe^2+^ form. The GSH content was assessed based on the intensity of the yellow color produced by its reaction with 5,5′-dithiobis(2-nitrobenzoic acid) (DTNB). The activity of GPx was assayed utilizing cumene hydroperoxide (Cum-OOH) as the substrate, with 5,5′-dithiobis(2-nitrobenzoic acid) (DTNB) serving as the chromogenic agent. The formation of MDA was evaluated using thiobarbituric acid (TBA) as a reactive substrate at 532 nm. The protein content in liver, gill, muscle, and intestine tissues was quantified using the bicinchoninic acid (BCA) assay and the OD value was measured at 562 nm. During the data analysis, the NC (normal control) group served as the control.

### 2.3. Determination of Amino Acids and Fatty Acids in Muscle

The wet muscle tissue (100 mg) was added to 10 mL of 6 mol/L hydrochloric acid solution. The mixture was then subjected to hydrolysis at 110 °C for 22 h. Following hydrolysis, the resulting hydrolysate was filtered into a 50 mL volumetric flask. Next, 1.0 mL of the filtrate was transferred into a 15 mL test tube and concentrated under reduced pressure at 40 °C. After drying, the concentrated mixture was dissolved using 1.0 mL of sodium citrate buffer (pH 2.2). Finally, the solution was filtered through a 0.22 μm filter membrane to analyze amino acids using automatic amino acid analyzer (HITACHI, Japan).

The muscle tissue (200 mg) was hydrolyzed by adding 10 mL of HCl (8.3 M) at 70–80 °C for 40 min. The resulting hydrolysate was used for total lipid extraction by adding 30 mL of a mixture of diethyl ether and petroleum ether (1:1, *v*/*v*) according to the method in the national standard of China (GB5009.168–2016) [33]. To convert the extracted lipid into methyl esters, it was subjected to methyl esterization using a 14% boron trifluoride–methanol solution at 45 °C for 20 min. The fatty acid methyl esters (FAMEs) were then analyzed using a gas chromatography instrument (Agilent 7890A, Agilent Technologies, Santa Clara, CA, USA) equipped with an HP-88 Agilent column (100 m × 0.25 mm × 0.20 μm). The injector and detector temperatures were set at 250 °C and 260 °C, respectively. The fatty acid composition was identified by comparison with 37 kinds of FAME standards (Sigma, St. Louis, MO, USA).

### 2.4. Non-Targeted Metabolome Sequencing in Muscle

The muscle metabolites were extracted using a solution (acetonitrile: methanol = 1:1) via centrifugation (12,000 rpm, 15 min, and 4 °C) from the NC group (named MNC, 3 pooled samples) and the 0.8 g/kg Pro group (named MPro, 3 pooled samples). The resulting supernatant was analyzed using an UHPLC system (Vanquish, San Jose, CA, USA) and a QE-MS (Orbitrap MS, San Jose, CA, USA). The raw data were converted into the mzXML format and metabolite annotation was performed using an in-house MS2 database (BiotreeDB, V2.1). To improve metabolite coverage, the metabolites were detected in both positive and negative ion modes. Principal component analysis (PCA) was used to evaluate the preliminary differences between groups of samples. Orthogonal projection to latent structures-discriminant analysis (OPLS-DA) was applied to distinguish the metabolomics profile of the two groups. The OPLS-DA model was further evaluated through cross-validation and permutation tests. Differential metabolites between the NC and 0.8 g/kg Pro groups were identified by comparing the VIP score of the OPLS-DA model using the following threshold values: VIP score ≥ 1 and *p*-value < 0.05 (*t*-test). The differential metabolites were mapped to the KEGG database to identify significantly enriched metabolic pathways.

### 2.5. Transcriptome Sequencing in Intestines

Total RNA was extracted from the intestinal tissue of the NC (4 pooled samples) and 0.8 g/kg Pro (4 pooled samples) groups using the TRIzol reagent kit (Invitrogen, San Diego, CA, USA) according to the manufacturer’s instructions. The mRNA was reverse-transcribed into cDNA after enrichment and fragmentation. The purified double-stranded cDNA was used to construct a library via PCR amplification, which was sequenced using Illumina Nova 6000 system (Gene Denovo, Guangzhou, China).

To obtain clean data, the raw data were filtered using fastp (version 0.18.0). After the removal of residual ribosomal RNA, the clean data were mapped to the reference genome of *Cyprinus carpio* (NCBI: GCF_000951615.1) using HISAT2. 2.4. PCA was performed to evaluate the distance relationship between samples. DESeq2 software (version 3.0) was used to identify the differentially expressed genes (DEGs) between the NC and Pro groups using the following threshold values: FDR < 0.05 and |log FC| ≥ 1. To identify biological functions and key signaling pathways, the DEGs were mapped onto the GO (gene ontology) and KEGG databases. Furthermore, we performed a gene set enrichment analysis (GSEA) to discover distinctive pathways and GO terms between the NC and Pro groups, and threshold values for significance were set as a |normalized enrichment score (ES)| > 1, a nominal *p*-value < 0.05, and an FDR < 0.25.

The transcriptome sequencing was further validated via quantitative real-time PCR (qPCR) analysis, with the specific primers utilized in this study listed in Table S2. Total RNA was isolated from intestinal tissue using RNAiso Plus reagent (Takara, Beijing, China). The RNA was then used to synthesize cDNA via reverse transcription using the PrimeScript™ RT reagent kit (Takara). The cDNA served as a template to perform qPCR using a TB Green Premix Ex Taq II kit (Takara, RR820A). The resulting Cq value was used to calculate the relative expression of each gene using the 2^−∆∆Cq^ method, with β-actin used as the housekeeping gene.

### 2.6. 16S rRNA Sequencing in Intestinal Bacteria

Microbial DNA from the intestinal content of fish in the NC (4 pooled samples) and 0.8 g/kg Pro (4 pooled samples) groups was isolated using HiPure Stool DNA Kits (Meiji Biotechnology, Guangzhou, China) in accordance with the manufacturer’s protocols. The target region of 16S rDNA was amplified by PCR using V3–V4 region primers (341F: CCTACGGGNGGCWGCAG, 806R: GGACTACHVGGGTATCTAAT). The amplicons were purified using AMPure XP Beads (Axygen, Union City, CA, USA), quantified using Real-Time PCR System (ABI, Foster City, CA, USA), and sequenced on an Illumina platform.

The raw data were subjected to a series of preprocessing steps, including merging, filtering, dereplication, denoising, and chimera removal, using the DADA2 R package (version 1.14). Following these procedures, the resulting clean tags were utilized to output the ASVs. The representative ASV sequences were classified into bacterial taxonomy using the RDP classifier (version 2.2) with reference to the SILVA database. After ASV annotation, the abundance statistics of each taxonomy were visualized using Krona (version 2.6). Alpha indices, including Chao1, Shannon, and Simpson, were calculated using the QIIME software (version 1.9.1), and the difference in these indices between the NC and 0.8 g/kg Pro groups was assessed by the Wilcoxon rank test. Principal coordinates analysis (PCoA) based on weighted Unifrac distances was plotted in R project, and the Anosim test was conducted using the Vegan package (version 2.5.3).

### 2.7. Statistical Analysis

SPSS was used to analyze the data in this study and the results are presented as the mean ± standard error of the mean (SEM). Differences in antioxidant parameters and growth parameters among groups were analyzed using ANOVA, followed by the LSD test. Differences in amino acid and fatty acid composition between the NC and Pro groups were analyzed using a *t*-test. The correlation between qPCR data and RNA-seq data was determined using the Pearson test. The level of significance was set at *p* < 0.05.

## 3. Results

### 3.1. Common Carp Growth Performance

During the experiment, one fish died in each of the NC, 0.2 g/kg Pro, and 0.8 g/kg Pro groups, while no fish died in the 0.4 g/kg Pro group (Table 1). After 10 weeks of feeding, there was a significant increase in the final weight and specific growth rate, but a significant decrease in the feed conversion ratio, in the groups fed 0.4 and 0.8 g/kg Pro compared to the NC group (*p* < 0.05; Table 1).

### 3.2. Antioxidant and Lipid Peroxidation Parameters in Different Tissues

**Figure 1:**
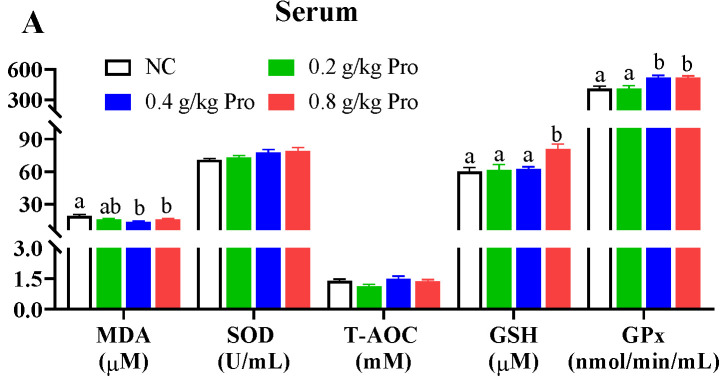

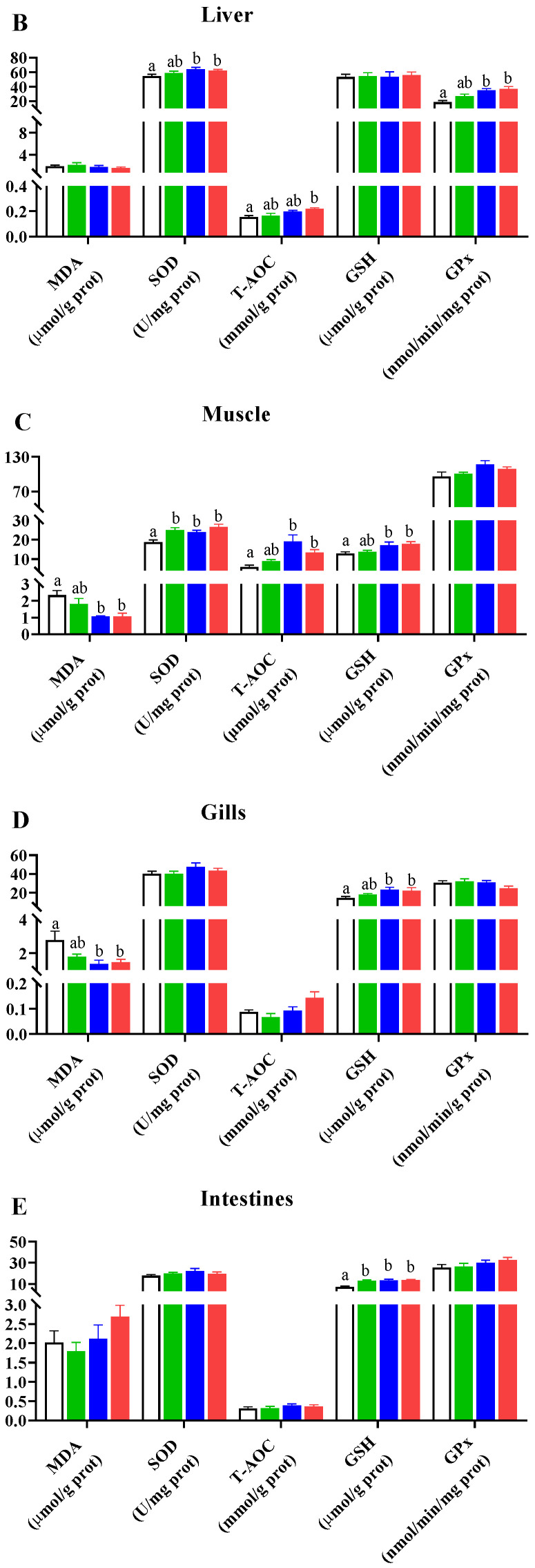
Effects of dietary Pro on antioxidant capacity of *Cyprinus carpio* after 10 weeks of farming. (**A**–**E**) Antioxidant parameters in the serum, liver, muscle, gill, and intestine, respectively. The results are expressed as the mean ± SEM (n = 9). Different letters above the bars indicate significant differences for each parameter between groups (*p* < 0.05).

Antioxidant capacity was assessed by measuring the levels of MDA, SOD, T-AOC, GSH, and Gpx in different tissues (Figure 1). In the serum (Figure 1A), following Pro treatment, MDA content exhibited a declining trend, with significant reductions observed in the groups supplemented with 0.4 and 0.8 g/kg of Pro compared to the NC group (*p* < 0.05). Conversely, Gpx activity displayed an increasing trend, and it was significantly enhanced in the 0.4 and 0.8 g/kg Pro-supplemented groups relative to the NC group (*p* < 0.05). The GSH content also showed an increasing trend and a significant increase was observed in the 0.8 g/kg Pro-supplemented group relative to the NC group (*p* < 0.05). However, the SOD and T-AOC levels were not impacted by dietary Pro supplementation (*p* > 0.05).

In the liver (Figure 1B), following Pros administration, levels of SOD, Gpx, and T-AOC uniformly demonstrated an upward trend. For SOD and Gpx activities, there were significant differences in the groups supplemented with 0.4 and 0.8 g/kg Pro compared to the NC group (*p* < 0.05). Additionally, the T-AOC level was significantly higher in the 0.8 g/kg Pro-supplemented group than in the NC group (*p* < 0.05). However, there was no change in MDA and GSH levels among the different groups (*p* > 0.05).

In the muscle (Figure 1C), Pro treatment improved the levels of SOD, T-AOC, and GSH and lowered the MDA content. Notably, there was a marked decrease in the MDA content and a marked increase in the levels of SOD, T-AOC, and GSH in the groups fed 0.4 and 0.8 g/kg Pro compared with the NC group (*p* < 0.05). A similar increase in SOD level was also observed in the 0.2 g/kg Pro-treated group (*p* < 0.05). However, Gpx activities were not significantly changed by Pro treatment.

In the gills (Figure 1D), Pro treatment inhibited MDA formation but enhanced GSH production. Significant changes were observed in the 0.4 and 0.8 g/kg Pro treatments compared to the NC treatment (*p* < 0.05). Moreover, the levels of SOD, T-AOC, and Gpx were not altered by dietary Pro feeding (*p* > 0.05).

In the intestines (Figure 1E), the content of GSH was significantly increased in the three Pro-treated groups compared to the NC group (*p* < 0.05). However, no significant differences were observed in other parameters among the different treatments (*p* > 0.05).

### 3.3. Amino Acid and Fatty Acid Composition in Muscle

There were 17 amino acids identified in the muscle tissue of common carp, including 9 essential amino acids (EAAs) and 8 non-essential amino acids (NEAAs) (Table S3). EAAs consisted of Thr, Val, Met, Ile, Leu, Phe, Lys, His, and Arg, whereas NEAAs included Asp, Ser, Glu, Gly, Ala, Gys, Tyr, and Pro. However, there were no differences in the levels of these amino acids between the NC and 0.8 g/kg Pro groups.

Eleven fatty acids were detected in the muscle tissue of common carp, including two saturated fatty acids (SFA) and nine unsaturated fatty acids (NFA) (Table 2). The levels of C16:0, C18:0, C18:3n3, C20:1, C20:2, C22:1n9, and C20:4n6 were significantly higher in the 0.8 g/kg Pro group than in the NC group (*p* < 0.05). Similarly, Pro treatment exhibited higher levels of total polyunsaturated fatty acids (PUFA), including n-3 and n-6 PUFA (*p* < 0.05). Additionally, the n-6/n-3 ratio was slightly reduced, while the PUFA/SFA ratio was slightly increased in the Pro group compared to the NC group.

### 3.4. Metabolomics Analysis in Muscle

One sample from both the NC group and the 0.8 g/kg Pro group was excluded during metabolome sequencing due to sample degradation; thus, the metabolomic analysis was conducted with three pooled samples from each group. Quality control (QC) analysis showed that the sequencing data were acceptable based on the well-clustered QC samples (Figure S2). In the muscle, we identified 3086 metabolites in positive ion mode and 3025 metabolites in negative ion mode (Figure S3A,B). The unsupervised PCA displayed a clear separation between the NC and 0.8 g/kg Pro groups in both positive and negative ion modes (Figure S3C,D). The OPLS-DA results also showed that the metabolites between the NC and 0.8 g/kg Pro groups exhibited different classifications in both positive and negative (Figure S3E,F) ion modes. In addition, cross-validation and permutation test results revealed that the OPLS-DA model for the metabolites was reliable (Figure S3G,H).

**Figure 2:**
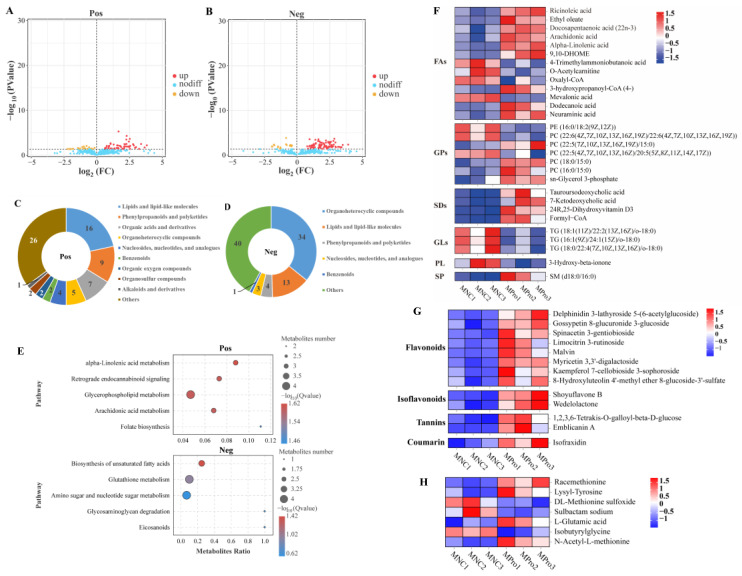
Differential metabolites in the muscle of *C. carpio* between the NC (MNC) and 0.8 g/kg Pro-fed groups (MPro). (**A**,**B**) Volcano plot of the differential metabolites in positive (Pos) and negative (Neg) ion modes. (**C**,**D**) Numbers and classification of the differential metabolites in Pos and Neg ion modes. (**E**) Main KEGG pathways enriched by the differential metabolites in Pos and Neg ion modes. (**F**) Differential metabolites related to lipids and lipid-like molecules (FA, fatty acyls; GP, glycerophospholipids; SD, steroids and steroid derivatives; GL, glycerolipids; SP, sphingolipid; and PL, prenol lipid). (**G**) Differential metabolites related to phenylpropanoids and polyketides. (**H**) Differential metabolites related to amino acids.

Dietary 0.8 g/kg Pro supplementation resulted in an increase in 53 metabolites and a decrease in 21 metabolites in positive ion mode, while it led to an increase in 78 metabolites and a decrease in 17 metabolites in negative ion mode, compared to the NC group (Figure 2A,B). In positive ion mode (Figure 2C), the differential metabolites primarily belonged to lipids and lipid-like molecules (16), phenylpropanoids and polyketides (8), and organic acids and derivatives (7). In negative ion mode (Figure 2D), the differential metabolites were mainly organoheterocyclic compounds (34), lipids and lipid-like molecules (13), and phenylpropanoids and polyketides (4). KEGG enrichment analysis revealed that the differential metabolites were primarily associated with α-linolenic acid (α-LA) metabolism (*q* value = 0.024), glycerophospholipid (GP) metabolism (*q* value = 0.025), arachidonic acid (ARA) metabolism (*q* value = 0.025), and biosynthesis of UFA (*q* value = 0.038) (Figure 2E).

The analysis of lipid-related metabolites revealed significant alterations in 13 fatty acyls (FAs), 7 GPs, 4 steroids and steroid derivatives (SDs), 3 glycerolipids (GLs), 1 sphingolipid (SP), and 1 prenol lipid (PL) between the NC and 0.8 g/kg Pro groups (Figure 2F). Among FAs, nine metabolites (e.g., ARA, α-LA, ricinoleic acid, and docosapentaenoic acid) were significantly increased by 0.8 g/kg Pro treatment, while four metabolites were significantly decreased (*p* < 0.05). In GPs, 0.8 g/kg Pro treatment resulted in a marked increase in the levels of PC (16:0/15:0), PC (22:5/15:0), and PC (18:0/15:0), but a decrease in the levels of PC (22:6/22:6) and PC (22:5/20:5) (*p* < 0.05). In GLs, the three types of triglyceride (TG) levels were suppressed by 0.8 g/kg Pro treatment (*p* < 0.05).

Furthermore, 0.8 g/kg Pro treatment significantly increased the levels of various bioactive compounds, including eight flavonoids, two isoflavonoids, two tannins, and one coumarin (Figure 2G; *p* < 0.05). As shown in Figure 2H, Pro treatment also resulted in alterations in several amino acid derivatives. In detail, racemethionine (DL-methionine), lysyl-tyrosine, L-glutamic acid, and N-acetyl-L-methionine increased, while DL-methionine sulfoxide, sulbactam sodium, and isobutyrylglycine decreased, compared to the NC group (*p* < 0.05).

### 3.5. Transcriptomic Analysis in Intestines

The transcriptomic analysis was performed using four pooled samples from each group. After filtering, transcriptome sequencing obtained a total of 5,392,441,669–7,005,632,038 bp of clean reads (Table S4). The quality control results indicated that the sequencing data obtained from the intestines of the NC and 0.8 g/kg Pro groups were highly reliable (Table S4). The PCA demonstrated a distinct separation between the NC and 0.8 g/kg Pro groups, indicating that Pro treatment had a significant impact on the gene expression patterns in the intestinal tissue (Figure S4A). A total of 1909 DEGs were identified between the NC and 0.8 g/kg Pro groups, with 1035 upregulated genes and 874 downregulated genes in the Pro group compared to the NC group (Figure S4B,C).

To gain a deeper understanding of the effects of Pro treatment on biological function in the intestines, we conducted a GO enrichment analysis, focusing on three main categories: molecular function, cellular component, and biological process (Figure S5A). In the biological process, the DEGs were highly associated with lipid metabolic process (*p*.adjust < 0.001) and anion transport (*p*.adjust < 0.001) (Figure S5B). In the molecular function, the DEGs were primarily involved in exopeptidase activity (*p*.adjust < 0.001) and organic acid transmembrane transporter activity (*p*.adjust < 0.001) (Figure S5C). In the cellular component, the DEGs were primarily enriched in lysosome (*p*.adjust < 0.001) and vacuole (*p*.adjust < 0.001) (Figure S5D).

**Figure 3:**
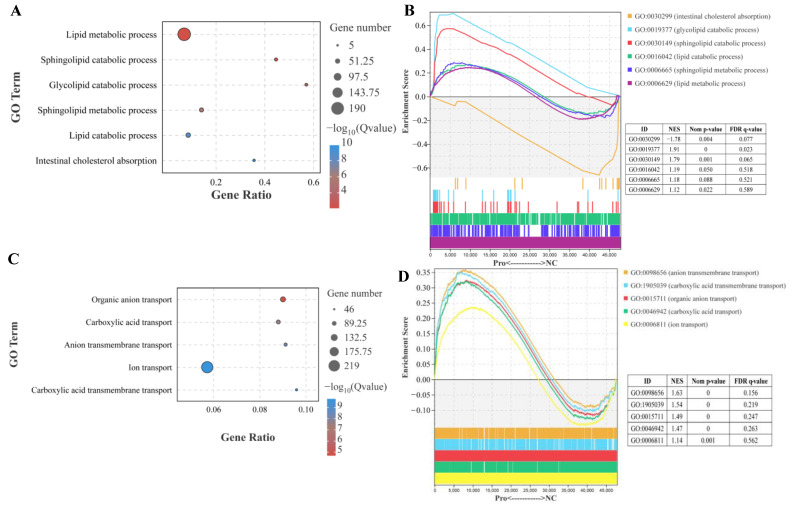
Effects of dietary Pro supplementation on lipid metabolism and ion transport in the intestines of *C. carpio*. (**A**) Differential GO terms related to lipid metabolism. (**B**) GSEA for the GO terms related to lipid metabolism. (**C**) Differential GO terms related to ion transport. (**D**) GSEA for the GO terms related to ion transport.

In the lipid metabolic process, sphingolipid catabolic process, glycolipid catabolic process, and lipid catabolic process were significantly affected by dietary 0.8 g/kg Pro supplementation (*p*.adjust < 0.001; Figure 3A). Furthermore, GSEA showed that these processes exhibited a high enrichment in 0.8 g/kg Pro group. Specifically, the sphingolipid catabolic process and glycolipid catabolic process showed statistically significant differences (Figure 3B). However, intestinal cholesterol absorption exhibited significantly lower enrichment in 0.8 g/kg Pro treatment (Figure 3A,B). In GO terms related to ion transport, specifically, organic anion transport, carboxylic acid transport, anion transmembrane transport, and carboxylic acid transmembrane transport were noticeably altered by 0.8 g/kg Pro treatment (Figure 3C). Further analysis using GSEA confirmed that these processes exhibited a higher enrichment in the 0.8 g/kg Pro group, with a significant difference in organic anion transport, anion transmembrane transport, and carboxylic acid transmembrane transport (Figure 3D).

**Figure 4:**
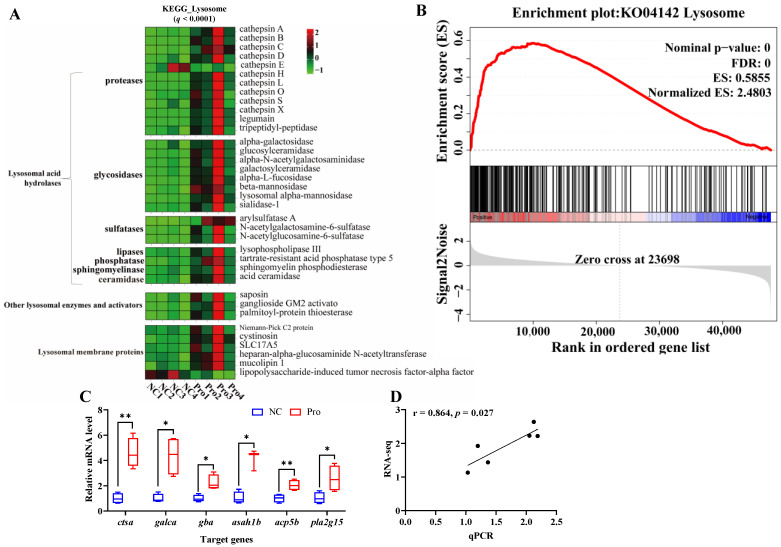
Changes in the lysosome pathway in the intestines of *C. carpio* between the NC and Pro groups. (**A**) DEGs in the KEGG lysosome pathway. (**B**) GSEA for the KEGG lysosome pathway. (**C**) Key genes expression in the lysosome pathway measure by qPCR, with values expressed as the mean ± SEM (n = 4), * *p* < 0.05 and ** *p* < 0.01. (**D**) Correlation between the qPCR and RNA-seq data.

To further investigate the key signaling pathways, we performed KEGG enrichment analysis using the DEGs (Figure S6). The DEGs were found to be enriched in five KEGG A classes: metabolism, organismal systems, cellular processes, genetic information processing, and environmental information processing (Figure S6A). The top 10 pathways were primarily associated with lysosome and metabolism function, specifically, sphingolipid metabolism and glycosphingolipid biosynthesis (Figure S6B,C). In the lysosome pathway, 34 genes were upregulated and 2 genes were downregulated following 0.8 g/kg Pro treatment (*q* < 0.0001; Figure 4A). GSEA confirmed the strong enrichment of the lysosome pathway in the 0.8 g/kg Pro group (Figure 4B). Further, we validated the expression of key genes involved in the lysosome pathway, including *ctsα*, *galcα*, *gba*, *asah1b*, *acp5b*, and *pla2g15*, using qPCR (Figure 4C), and the data revealed a clear positive correlation with the RNA-seq results (r = 0.864, *p* = 0.027; Figure 4D).

**Figure 5:**
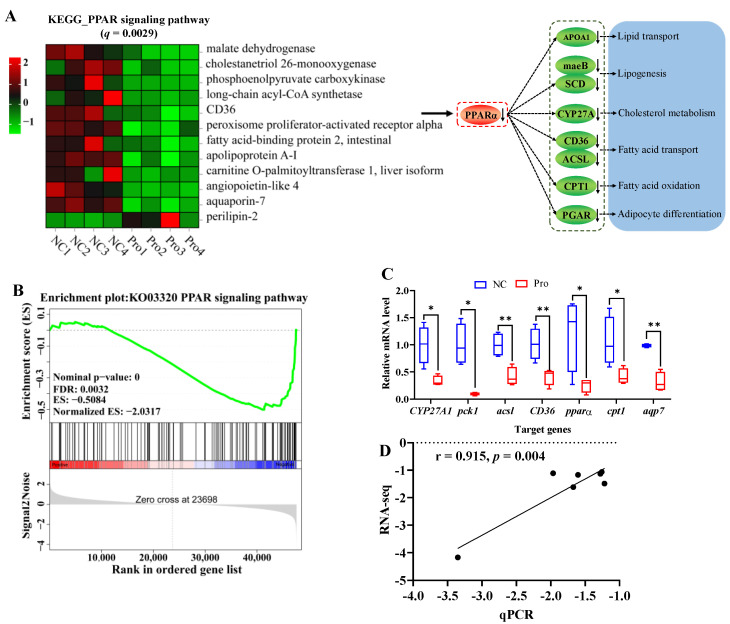
Changes in the PPAR signaling pathway in the intestines of *C. carpio* between the NC and Pro groups. (**A**) DEGs in the PPAR signaling pathway and possible mechanism regulating lipid metabolism. (**B**) GSEA for the PPAR signaling pathway. (**C**) Key genes expression in the PPARα signaling pathway measure by qPCR, with values expressed as the mean ± SEM (n = 4), * *p* < 0.05 and ** *p* < 0.01. (**D**) Correlation between the qPCR and RNA-seq data.

It is important to highlight that 0.8 g/kg Pro treatment had a significant impact on the PPAR signaling pathway (*q* = 0.0029, Figure 5A). Specifically, 11 genes including *pparα* (a master in the PPARα signaling pathway) were downregulated, while 1 gene was upregulated, following 0.8 g/kg Pro treatment. Meanwhile, the GSEA results also indicated that the PPAR signaling pathway tended to be downregulated in the 0.8 g/kg Pro group (Figure 5B). Furthermore, the qPCR results showed a significantly positive correlation with RNA-seq, indicating the credibility of the transcriptome results (r = 0.915, *p* = 0.004; Figure 5C,D).

### 3.6. Intestinal Microbiota Characteristics

Microbiota characteristics were analyzed using four pooled samples from each group. Venn diagram analysis indicated that the total number of ASVs in the two groups was 6049 (Figure S7A). The Pro group had a higher total number of ASVs (3618) compared to the NC group (2899) (*p* > 0.05). Furthermore, there were 468 ASVs that were shared between the NC and 0.8 g/kg Pro groups. At the phylum level, 23 out of the total 29 phyla were found to be common to both the NC and 0.8 g/kg Pro groups (Figure S7B). At the genus level, 167 out of 336 genera were shared between the NC and 0.8 g/kg Pro groups (Figure S7C).

**Figure 6:**
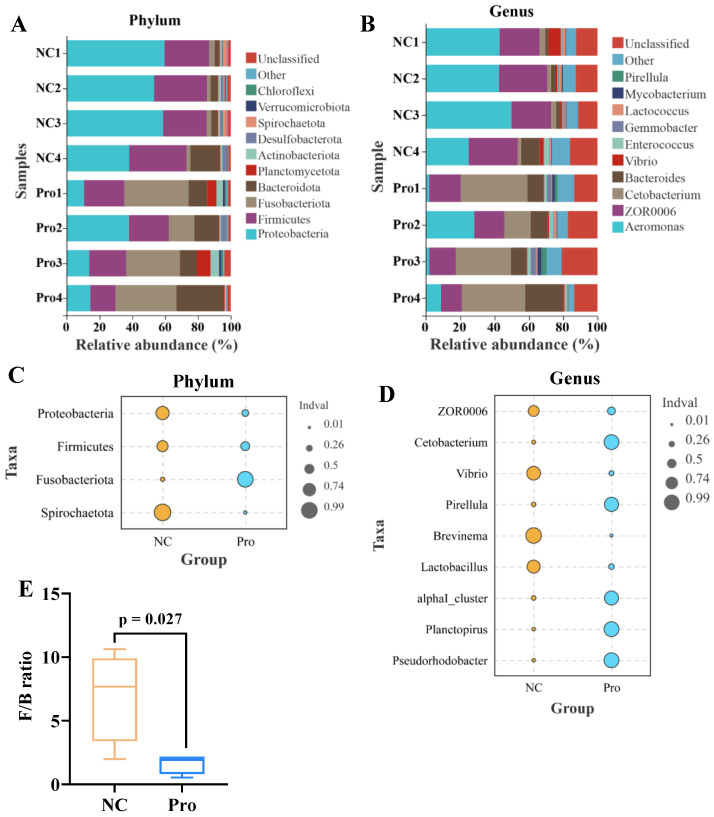
The proportions of intestinal microbiota in the NC and Pro groups. (**A**,**B**) The top 10 phyla and genera of intestinal microbiota in the two groups. (**C**,**D**) Differential phyla and genera in intestinal microbiota between the NC and Pro groups (Wilcoxon test, *p* < 0.05). (**E**) Firmicutes/Bacteroidetes (F/B) ratio in the intestinal microbiota collected from the NC and Pro groups.

Microbial composition analysis revealed that the five most predominant phyla in the NC group were Proteobacteria (52.34%), Firmicutes (30.26%), Bacteroidota (6.97%), Fusobacteriota (2.73%), and Desulfobacterota (1.77%) (Figure 6A). In contrast, the 0.8 g/kg Pro group displayed a different pattern, with the top five phyla being Fusobacteriota (31.08%), Firmicutes (21.84%), Proteobacteria (18.93%), Bacteroidota (16.39%), and Planctomycetota (3.81%) (Figure 6A). At the genus level, *Aeromonas* (39.94%), *ZOR0006* (25.89%), *Bacteroides* (4.14%), *Vibrio* (2.97%), and *Cetobacterium* (2.58%) were the top five microbes in the NC group, while *Cetobacterium* (30.85%), *ZOR0006* (15.98%), *Bacteroides* (12.66%), *Aeromonas* (10.01%), and *Enterococcus* (1.83%) dominated in the 0.8 g/kg Pro group (Figure 6B).

Indicator species analysis showed that Fusobacteriota has a higher relative abundance, while Proteobacteria, Firmicutes, and Spirochaetota exhibited lower relative abundance in the 0.8 g/kg Pro group, compared to the NC group (Figure 6C). Similarly, at the genus level, the relative abundance of *Cetobacterium*, *Pirellula*, *alphaI_cluster*, *Planctopirus*, and *Pseudorhodobacter* were higher, whereas the relative abundance of *ZOR0006*, *Vibrio*, *Brevinema*, and *Lactobacillus* were lower in the 0.8 g/kg Pro group, compared to the NC group (Figure 6D). Notably, the Firmicutes/Bacteroidetes (F/B) ratio showed a decrease in the 0.8 g/kg Pro group compared with the NC group (Figure 6E).

**Figure 7:**
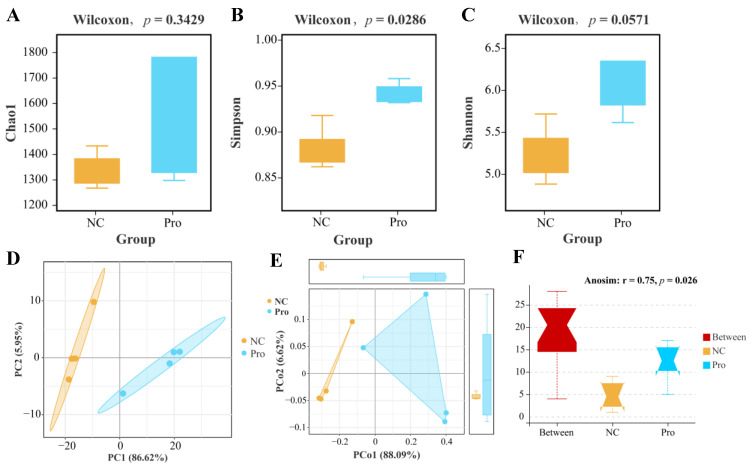
The α diversity (**A**–**C**) and β diversity (**D**–**F**) analysis of intestinal microbiota between the NC and Pro groups. (**A**) Chao1 index. (**B**) Simpson’s index of diversity. (**C**) Shannon index. (**D**) PCA score plot. (**E**) PCoA score plot. (**F**) Anosim test.

According to α-diversity analysis, we observed a non-significant elevation in the Chao1 and Shannon indices (*p* > 0.05; Figure 7A,B) and a significant increase in the Simpson’s index of diversity (*p* < 0.05; Figure 7C) in the 0.8 g/kg Pro group compared to the NC group. As for β-diversity, both PCA and PCoA analyses indicated that the samples of the NC and 0.8 g/kg Pro groups showed a distinct cluster (Figure 7D,E). Furthermore, Anosim analysis revealed a significant difference in the microbial community composition between the NC and 0.8 g/kg Pro groups (r = 0.75, *p* = 0.026; Figure 7F).

**Figure 8:**
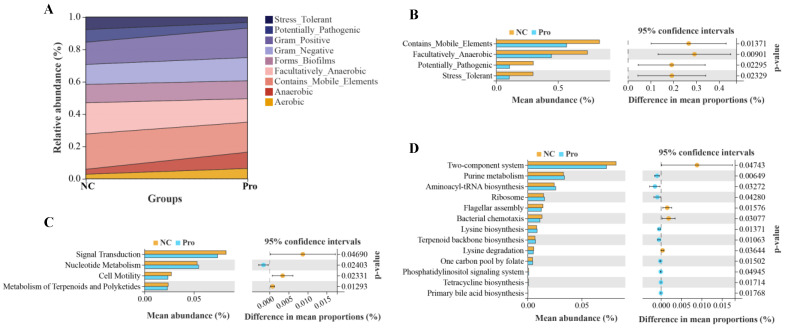
The analyses for potential phenotypes and functions of intestinal microbiota in the NC and Pro groups. (**A**) Relative abundance of nine potential phenotypes of intestinal microbiota. (**B**) Differential phenotypes of the intestinal microbiota between the NC and Pro groups. (**C**) Differential pathways at level 2 KEGG between the NC and Pro groups. (**D**) Differential pathways at level 3 KEGG between the NC and Pro groups. Statistical significance was identified by Welch’s *t*-test with *p* < 0.05.

We further predicted and analyzed nine potential bacterial phenotypes in the NC and 0.8 g/kg Pro groups (Figure 8A). Compared with the NC group, the relative abundance of mobile element containing, facultatively anaerobic, potentially pathogenic, and stress-tolerant bacteria were significantly lower in the intestine of the 0.8 g/kg Pro group (*p* < 0.05; Figure 8B). Tax4Fun analysis revealed that in the level 2 KEGG pathway, signal transduction, cell motility, and metabolism of terpenoids and polyketides were decreased in the 0.8 g/kg Pro group compared to the NC group (*p* < 0.05; Figure 8C). At the level 3 KEGG pathway, several pathways were found to be enhanced in the 0.8 g/kg Pro group, including purine metabolism, aminoacyl-tRNA biosynthesis, terpenoid backbone biosynthesis, tetracycline biosynthesis, and primary bile acid biosynthesis (*p* < 0.05; Figure 8D), suggesting that Pro mediated microbe–microbe and microbe–host interactions.

### 3.7. Interactions between Intestinal Microbes and Lipid Metabolism

**Figure 9:**
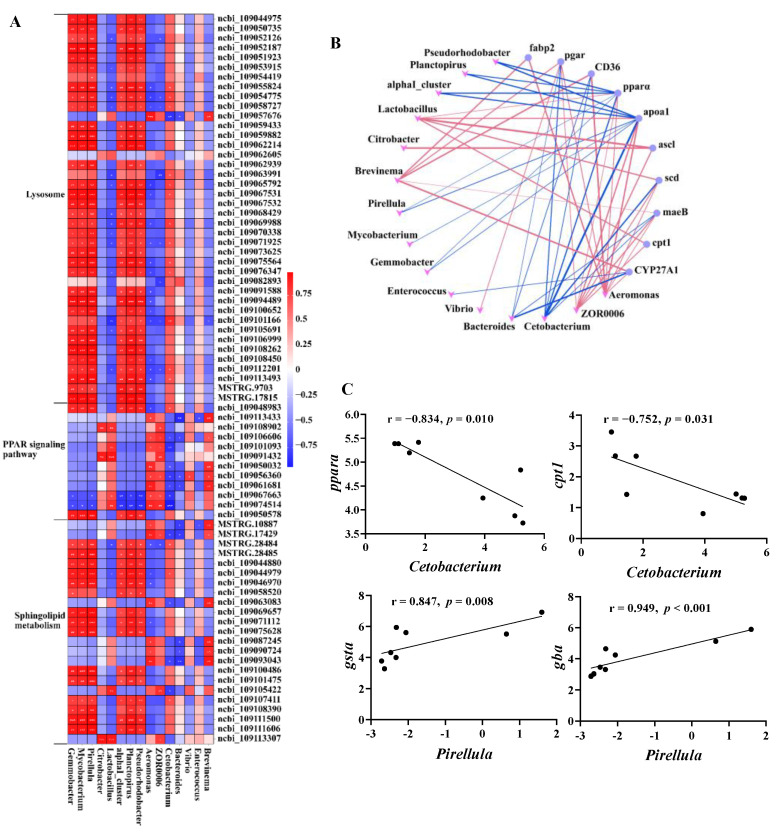
Interactions between the intestinal microbes and DEGs related to lipid metabolism. (**A**) Correlation heatmap of microbe–DEGs. (**B**) Network plot of significant microbe–gene (PPAR signaling pathway) correlations; red lines indicate positive correlation and blue lines indicate negative correlation. (**C**) Correlation plots of representative gene–microbe combinations.

To investigate the relationship between microbes and host genes in the intestines of common carp after 0.8 g/kg Pro feeding, we conducted a correlation analysis between 75 DEGs enriched in lipid metabolism and 15 microbial taxa at the genus level (Figure 9). As shown in Figure 9A, we observed significant positive correlations (*p* < 0.05) between the majority of DEGs involved in the lysosome pathway and sphingolipid metabolism and the relative abundance of *Gemmobacter*, *Mycobacterium*, *Pirellula*, and *Parabacteroides*. Additionally, several notable negative correlations (*p* < 0.05) were observed between dominant taxa, such as *Aeromonas* and *ZOR0006*, and specific genes associated with the lysosome pathway, including *cystinosin*, *lysosomal alpha-mannosidase*, and *acid ceramidase*.

We also specifically investigated the correlation between microbiota and the PPAR pathway, and visually depicted the significant gene–microbe correlations (*p* < 0.05; Figure 9B). The results demonstrated a statistically significant positive correlation between predominant taxa, specifically, *Aeromonas* and *ZOR0006*, and the majority of genes in the PPAR pathway. Conversely, a significant negative correlation was observed between *Cetobacterium*, which was significantly increased in the 0.8 g/kg Pro group, and the majority of genes.

In addition to the overall network, Figure 9C illustrates the correlation between representative gene expression and microbial taxa, both of which have previously been linked to host health and thus may be of interest. The *pparα*, a crucial regulator of lipid metabolism, was significantly negatively correlated with *Cetobacterium* (r = −0.834, *p* = 0.01). Similarly, *cpt1*, a vital enzyme involved in fatty acid oxidation, exhibited a negative correlation with *Cetobacterium* (r = −0.752, *p* = 0.031). Furthermore, both *gsta* (r = 0.874, *p* = 0.008) and *gba* (r = 0.949, *p* < 0.001) showed a positive correlation with *Pirllula*. CTSA (Cathepsin A) is a key proteolytic enzyme in the lysosome, while GBA (glucosylceramidase) is a crucial enzyme in the catabolic pathway for glucosylceramide, a membrane sphingolipid and a precursor for various glycolipids.

## 4. Discussion

Pros are a type of polyphenolic compounds known for their potent antioxidant activity. They are abundantly present in various plant sources, such as grape seed, pinto bean, and blueberry. Emerging research has highlighted the important role of Pros in promoting animal growth, maintaining health, and preventing disease. Nevertheless, the effects of Pros on growth performance, muscle quality, and nutrients, as well as intestinal microbiota and function, may vary across different animal species.

### 4.1. The Effect of Pros on Growth Performance

Pros have reportedly been used as appetite stimulators and growth promoters in cultured fish. Adding Pros to the diet improved weight gain and feed utilization in juvenile American eel, European eel, and tilapia [20,21,34]. In addition, dietary Pro has been shown to ameliorate the growth retardation caused by histamine or cadmium stress in American eel, pearl gentian grouper, and tilapia [22,23,35]. In line with previous studies, the present study found that the common carp fed with 0.4 and 0.8 g/kg Pro exhibited higher growth performance, suggesting that Pro, as a feed additive, can effectively promote the growth of common carp. At present, the mechanism underlying the beneficial effect of Pros on fish growth remain unknown, but potential explanations have been mentioned in previous studies. Some studies have suggested that Pros can enhance the activities of intestinal digestive enzymes, thereby improving feed utilization rate [36,37]. Furthermore, other studies indicated that Pros could regulate intestinal microbial community composition, maintain intestinal health, and promote nutrient absorption, ultimately enhancing growth [38].

### 4.2. The Effect of Pros on Antioxidant Capacity

Animal experiments have shown that Pro treatment decreased the levels of reactive oxygen species (ROS) in different tissues or cells [10,39]. Pros also have the potential to improve cellular antioxidant systems, such as SOD, catalase, or Gpx systems [40]. The antioxidant properties of Pros could potentially lead to several beneficial effects, including anti-inflammatory, antimicrobial, anticarcinogenic, hypolipemic, and antihyperalgesic activities [10]. It has been reported that Pro treatment can effectively prevent the formation of H_2_O_2_, protein oxidation, and DNA damage in cells by enhancing antioxidant defense compounds, such as Gpx, SOD, catalase, and GSH [15]. In fish, Pro treatment has been found to increase antioxidant enzymes (e.g., SOD and Gpx) and non-enzymatic antioxidants (e.g., GSH) in the serum of European eels [34] and hybrid sturgeon [36]. Consistent with previous studies, our data also showed the increased levels of SOD (in liver and muscle), T-AOC (in liver and muscle), GSH (in serum, muscle, gills, and intestines), and Gpx (in liver) after 0.4 and 0.8 g/kg Pro feeding. These results demonstrate that Pros can significantly improve antioxidant ability in aquatic animals. Interestingly, in the muscle, almost all antioxidant parameters were enhanced after the administration of Pros.

The intrinsic antioxidant defense system of fish can be compromised under adverse conditions, resulting in the excessive production of ROS that cause lipid peroxidation via reacting with unsaturated fatty acids. Pros have been shown to scavenge free radicals and inhibit lipid peroxidation in the muscle of finishing pigs [41]. Similarly, in hybrid sturgeons, the administration of 50 and 100 mg/kg of Pro was found to effectively inhibit MDA formation [36]. Moreover, Pro treatment also mitigated the lipid peroxidation induced by Cd stress in the intestine of pearl gentian grouper [22]. In agreement with previous studies, our data showed that lipid peroxidation was inhibited by 0.4 and 0.8 g/kg Pro treatments. These results highlight the antioxidation ability of Pro to avoid lipid peroxidation. It is worth noting that Pro treatment was observed to effectively suppress lipid peroxidation in muscle, potentially leading to an enhancement in meat nutritional quality. Low lipid peroxidation may suppress the degradation of PUFA and vitamins, and formation of harmful substances [42].

### 4.3. The Effect of Pros on Muscle Nutrient Quality

The muscle of fish is the primary edible portion and a valuable source of nutriment for humans. Amino acid composition is a vital determinant of fish muscle nutritional quality. The addition of Pros to the diet was found to increase the crude protein content of the body in American eel [21] and tilapia [20], but the impact of Pros on amino acid composition in fish muscle remains unclear. This study showed that the amino acid composition including EAA and NEAA was not significantly changed in the muscle of common carp after Pro treatment. Similar effects of Pro were also observed in finishing pigs [41]. However, we found that dietary Pro supplementation significantly changed the levels of some amino acids analogs, such as increased DL-methionine and L-glutamic acid, in the muscle of common carp. DL-methionine is not only a vital source of methionine, but it also serves as a precursor to essential intermediates such as glutathione [43]. Glutamic acid was considered as a flavor-related amino acid, which would enhance the flavor of fish flesh [44]. Therefore, the increased levels of DL-methionine and L-glutamic acid may have a positive effect on flesh quality in the muscle of common carp.

The composition of fatty acids is also a crucial factor in evaluating the nutritional and health benefits of fish muscle. A high percentage of PUFAs in food has been linked to improved fetal development, brain health, and a reduced risk of coronary heart disease in humans [44]. In this study, the content of PUFAs, including both n-3 and n-6, in muscle was significantly increased by dietary supplementation of 0.8 g/kg Pro. More specifically, Pro treatment increased the levels of LA, docosapentaenoic acid (DPA), and ARA in the muscle. Similar findings were also observed in the muscle tissue of pigs treated with Pros [40]. n-3 PUFAs have been confirmed to possess properties that improve anti-inflammation, antioxidant capacity, and meat nutritional value [45]. LA, an essential fatty acid (EFA), exhibits cardiovascular-protective, neuro-protective, anti-osteoporotic, anti-inflammatory, and antioxidative effects [46]. ARA is also an EFA maintaining normal health, which plays a vital role in the functioning of all cells, especially in the nervous system, skeletal muscles, and immune system [47]. DPA is an important long-chain n-3 PUFA that can serve as a dietary source of eicosapentaenoic acid. It is also known to play a role in improving risk markers associated with cardiovascular and metabolic diseases [48]. Here, the increase in the levels of LA, DPA, ARA, and n-3 PUFAs suggests that dietary Pro supplementation may improve the nutritional and health benefits of common carp. Moreover, we found that α-LA metabolism, ARA metabolism, and biosynthesis of UFA pathways were significantly altered by Pro treatment, which may provide an explanation for the increase in the levels of these PUFAs.

PC and PE are the most abundant phospholipids in all types of mammalian cells and subcellular organelles, playing a crucial role in regulating lipid, lipoprotein, and energy metabolism [49]. They also acts as reservoirs for essential PUFAs like DHA and ARA [50]. Furthermore, changes in PC and PE are associated with the formation of volatile flavor compounds in muscle tissue [51]. In this study, metabolome analysis revealed an increase in the levels of PC (16:0/15:0), PC (22:5/15:0), and PC (18:0/15:0), but a decrease in the levels of PE (16:0/18:2) and PC (22:6/22:6) in muscle tissue after Pro treatment. We hypothesize that these changes in PC and PE levels may impact the nutritional value and volatile flavor of muscle, but the detailed mechanism regarding this phenomenon still requires further investigation. In addition, the decreased PE may prevent coalescence of the lipid droplets in muscle tissue [52].

Lipids are vital factors that affect meat quality, and excessive lipid content can reduce both meat quality and feed efficiency. Among the various types of lipids, triglycerides (TGs) are the most abundant in the muscle of many fishes [53]. Modifying TG content can reduce the risk of cardiovascular disease in consumers [54]. Previous studies have suggested that flavonoids can decrease the TG content of meat and increase the proportions of total PUFA in the breast muscle [55]. Consistent with these findings, the content of TGs, including TG (18:1/22:2/o-18:0), TG (16:1/24:1/o-18:0), and TG (18:0/22:4/o-18:0), in the muscle was reduced by Pro treatment. This reduction could potentially have positive impacts on the health of consumers who consume carp as a food. Animal models studies have demonstrated that Pros have a positive effect on the TG metabolism, such as reducing plasma TGs and controlling the endogenous liver lipid production (reviewed in [56]). Based on these findings, we hypothesize that this is a possible explanation for the decrease of TG levels in muscle tissue.

It is intriguing to note that the levels of 13 bioactive compounds, including 8 flavonoids, 2 isoflavonoids, 2 tannins, and 1 coumarin, significantly increase in the muscle of common carp after being fed a diet containing Pro. These bioactive compounds have been proven to possess health benefits, such as antioxidant, anti-inflammatory, neuroprotective, and hepatoprotective effects, in animals [57]. They can also improves meat quality by regulating lipid metabolism and antioxidant capacity [58]. The increase of bioactive compounds in common carp muscle may be attributed to the absorption, metabolism, and accumulation of Pro. It has been reported that Pros may undergo direct absorption in the proximal intestinal tract or be absorbed in the gastrointestinal tract after being metabolized by gut microbiota in mammals [59,60]. Absorbed Pros and their metabolites can be transported to other organs through the circulation, exerting health-promoting effects [38]. However, the mechanism of absorption and metabolism of Pros in fish remains unclear due to significant differences in intestinal structure between fish and mammals.

### 4.4. The Effect of Pros on Lipid Metabolism in Intestines

Intestinal barrier integrity is crucial for maintaining intestinal health, as it not only aids in nutrient absorption but also shields against harmful substances. Numerous studies have shown that Pros administration can ameliorate intestinal dysbiosis caused by dietary factors [61]. They have protective effects against inflammatory response in the intestinal barrier [62], and prevent metabolic syndrome by regulating intestinal function and energy metabolism [63]. Moreover, previous studies have reported that Pros administration regulates intestinal lipid homeostasis to improve cardiometabolic disorders [64]. Our study found that Pro treatment resulted in a high enrichment of lipid catabolic process, including glycolipid and sphingolipid catabolic processes, suggesting that Pro treatment may enhance lipid catabolic metabolism. Increasing lipid catabolic metabolism may inhibit the accumulation of lipids in the intestines and increase the availability of fatty acids in the body [65,66]. This process may also provide a source of energy and help maintain normal metabolic activity in intestines. Dietary polyphenols have been reported to improve glycolipid metabolism disorders in animals [67]. For instance, Xu et al., (2019) found that Pro treatment ameliorated intestinal barrier dysfunction induced by a high-fat diet in rats by modulating glycolipid digestion [68]. In this study, the upregulation of glycolipid catabolic process may indicate a beneficial effect of Pros in inhibiting cellular glycolipid accumulation. Sphingolipids are one of the most important membrane lipids, participating in the formation of membrane microdomains. However, the abnormal accumulation of sphingolipids in cells has been associated with metabolic disorders [69]. In this study, the upregulation in sphingolipid catabolic process may have an influence on cell signaling, cellular homeostasis, and immune regulation in intestines. Notably, the changes in the sphingolipid catabolic process were consistent with alterations in the lysosomal pathway, indicating that sphingolipids were possibly degraded via the lysosomal catabolic pathways [70]. Lysosomes, small organelles that contain various hydrolytic enzymes such as proteases, lipases, and nucleases, can promote lipid catabolism and transport [71]. Disruption of lysosome function is considered a key factor leading to metabolic derangement and neurodegeneration [72]. Therefore, the upregulation of the lysosomal pathway in this study indicates that Pro treatment may contribute to maintaining cellular lipid homeostasis and preventing metabolic diseases.

It has been confirmed that Pro has a hypolipidemic effect, and one of its possible mechanisms may be related to the delay of cholesterol and lipid absorption in intestines [56]. Pros supplementation reduced cholesterol absorption by increasing the excretion of neutral steroids and bile acids [73,74]. Treatment with red wine polyphenolics (containing Pros) has been found to reduce free cholesterol and total cholesterol in Caco-2 cells [75]. In this study, Pro treatment caused lower enrichment of gene expression in intestinal cholesterol absorption, suggesting that Pros interfered in this process. Meanwhile, we also observed a significant downregulation in the mRNA level of CYP27A (an important enzyme regulating cholesterol metabolism), suggesting that Pro treatment may exert inhibitory effects on cholesterol metabolism in the intestines.

It is worth noting that our results also indicated a downregulation of the PPARα signaling pathway after Pros administration, which may further affect lipid absorption. PPARα has been identified as a key regulator in lipid metabolism, involved in various processes including fatty acid transport, synthesis, and oxidation, as well as lipogenesis [76]. In Caco2 cells, Pro treatment repressed *pparα* and *acsl3* gene expression to decrease TG secretion [77]. However, contradictory results have also been reported, in which Pro treatment upregulated PPARa and CPT1 and downregulated ACC and SREBP1 to modify intestinal lipid homeostasis [64]. In this study, the decreased expression of *pparα* further inhibited its target genes, such as *apoa1*, *maeB*, *scd*, *CD36*, *acsl*, and *cpt1*, suggesting that lipogenesis, fatty acid transport, fatty acid oxidation, and lipid transport may be suppressed by Pro treatment in the intestines of common carp.

### 4.5. The Effect of Pros on Intestinal Microbiota

Intestinal microbiota is increasingly being linked to fish health, playing a crucial role in regulating intestinal immunity, nutrient absorption, and overall host health. Dietary Pro has been studied in relation to intestinal microbiota in animals, but the findings are inconsistent and contentious, possibly due to variations in Pros sources or types, as well as differences in animal models [37]. In a piglet model, Pro treatment increased the abundance and diversity of intestinal bacteria [78]. Similarly, Pro treatment improved the decrease in α-diversity induced by a high fat diet in C57BL/6J mice [79]. In this study, the Pro group had a higher number of ASVs, and a significantly higher Simpson’s index of diversity in intestinal bacteria, compared to the NC group. Meanwhile, in conjunction with the β-diversity analysis, we speculate that Pro treatment may enhance the diversity of the intestinal microbiota in common carp. Interestingly, we also found that Pro treatment altered the relative abundance in the top four phyla and genus of the intestinal microbiota and resulted in a more balanced distribution among dominant taxa.

In animal intestines, the Firmicutes and Bacteroidetes phyla are dominant and play essential roles in promoting host health, boosting immunity, and maintaining homeostasis [80]. An elevated ratio of F/B is linked with some metabolic syndrome, such as obesity and chronic inflammation [81]. A marked reduction in the ratio of F/B was observed in ovariectomized mice after treatment with Pros (grape seed extract) [82]. Moreover, Pro treatment has been shown to alter the gut microbiota by increasing Bacteroidetes and decreasing Firmicutes, effectively alleviating metabolic syndrome induced by a high-fat diet (reviewed by Redondo-Castillejo, et al. [83]). Consistent with previous findings, our data also revealed a significant decrease in Firmicutes abundance and a 2.35-fold increase (but *p* > 0.05) in Bacteroidota abundance in the Pro group. Meanwhile, the ratio of F/B was significantly decreased by Pro treatment. These data suggest that dietary Pro may prevent metabolic syndrome in the intestines of common carp. In addition, some studies have found that dysbiotic Proteobacteria expansion is associated with epithelial dysfunction and inflammation [83,84]. Our results showed a lower Proteobacteria abundance in the Pro group, suggesting that Pro may have potential effects against epithelial dysfunction and inflammation in intestines.

Further, we observed the impact of Pro on intestinal microbiota at the genus. Here, compared with the NC group, there was a higher relative abundance in *Cetobacterium* and *Pirellula*, and a lower relative abundance in *Vibrio*, at the genus level. *Cetobacterium* is a dominant taxa of the intestinal microbiota, being involved in maintaining fish health, enhancing nutrition, and providing protection against pathogenic bacteria [85,86]. *Pirellula*, an important bacterial genus in fish intestines, has the potential to act as a probiotic, positively influencing fish growth [87]. *Vibrio* is also an important and diverse genus of bacteria, being widely distributed in fish intestines and the aquatic environment. However, a specific group within the *Vibrio* genus consists of several highly pathogenic species, such as *V. anguillarum*, *V. harveyi*, and *V. alginolyticus,* that can cause diseases in aquatic animals [88]. Based on previously published results and our data, we hypothesize that the increase in *Cetobacterium* and *Pirellula*, coupled with the decrease in *Vibrio,* in the Pro group may indicate positive effects on the resistance of common carp to pathogenic bacteria infection. Of interest, our phenotype analysis also showed a significant decrease in potential pathogenicity in the Pro group. It is worth noting that the changes of the intestinal microbiota showed a significant correlation with intestinal lipid metabolism. Taken together, it can be deduced that dietary Pro may enhance disease resistance, as well as regulate intestinal lipid metabolism in common carp, by altering the diversity and abundance of intestinal microbiota. However, further research is needed to elucidate the detailed underlying mechanism. In addition, Pro treatment also resulted in changes in some microbiota at the genus level, such as *ZOR0006*, *Brevinema*, *Planctopirus*, and *Pseudorhodobacter*. However, the potential effects of these changes on the host remain uncertain, as there is limited knowledge regarding these bacteria.

## 5. Conclusions

In summary, dietary Pro supplementation improved growth performance, antioxidant ability, and muscle nutrients in common carp. Dietary Pro may regulate lipid absorption and accumulation by upregulating lipid catabolic metabolism, inhibiting cholesterol absorption, and downregulating the PPARα pathway in the intestines. Meanwhile, Pro supplementation improved intestinal microbiota diversity and induced alterations in intestinal microbial composition and functions. Specifically, the alterations in microbiota may participate in the regulation of lipid metabolism, enhance disease resistance, and improve muscle nutrients through the interaction between intestinal microbiota and host metabolism. Our study not only provides novel insights into the action of Pro in enhancing growth, improving muscle nutrients, and regulating lipid metabolism, but also strongly supports the application of Pro as a feed additive in aquaculture.

## Figures and Tables

**Table 1:** Effects of dietary Pro on growth performance of *Cyprinus carpio* after 10 weeks of farming.

Growth Index	NC	0.2 g/kg Pro	0.4 g/kg Pro	0.8 g/kg Pro
Initial body weight (g)	42.5 ± 1.71	42.6 ± 1.63	42.6 ± 1.89	40.5 ± 2.01
Final body weight (g)	89.9 ± 3.48 ^a^	86.5 ± 3.38 ^a^	101.5 ± 2.80 ^b^	103.5 ± 3.92 ^b^
Specific growth rate (%/d)	1.07 ± 0.05 ^a^	1.01 ± 0.06 ^a^	1.24 ± 0.04 ^b^	1.35 ± 0.01 ^b^
Feed conversion ratio	2.13 ± 0.02 ^a^	2.07 ± 0.02 ^a^	1.83 ± 0.04 ^b^	1.85 ± 0.03 ^b^
Survival ratio (%)	96.7	96.7	100	96.7

The results are expressed as the mean ± SEM. Different letters in the same line indicate significant differences among groups (*p* < 0.05). Specific growth rate (SGR) = 100 × [Ln (average final weight) − Ln (average initial weight)]/number of days, feed conversion ratio (FCR) = food consumption/biomass increment, and survival ratio = final number of fish/initial number of fish.

**Table 2:** Hydrolyzed fatty acid composition in muscle of *C. carpio* fed on normal diet (NC) and Pro-supplemented diet (Pro).

Fatty Acids (% of Total Fatty Acid)	Groups		
	NC	0.8 g/kg Pro	Sig.
C16:0	19.92 ± 0.41	22.41 ± 0.18	**
C18:0	7.72 ± 0.3	9.49 ± 0.15	**
C18:1n9c	22.67 ± 2.63	31.03 ± 2.79	ns
C18:2n6c	21.92 ± 2.05	29.5 ± 2.06	ns
C18:3n3	0.99 ± 0.04	1.72 ± 0.1	**
C20:1	1.04 ± 0.04	1.32 ± 0.07	*
C20:2	0.94 ± 0.08	1.20 ± 0.02	*
C20:3n6	2.53 ± 0.11	2.97 ± 0.13	ns
C22:1n9	0.84 ± 0.02	1.10 ± 0.03	**
C20:4n6	5.73 ± 0.35	7.81 ± 0.35	*
C22:6n3	3.24 ± 0.39	3.95 ± 0.11	ns
Total SFA	27.64 ± 0.7	31.91 ± 0.32	**
Total MUFA	24.55 ± 2.65	33.44 ± 2.84	ns
Total PUFA	35.35 ± 1.4	47.15 ± 1.81	**
n-3 PUFA	4.23 ± 0.41	5.67 ± 0.07	*
n-6 PUFA	30.18 ± 1.74	40.28 ± 1.73	*
n-6/n-3	7.61 ± 1.3	7.09 ± 0.24	ns
PUFA/SFA	1.28 ± 0.06	1.48 ± 0.08	ns

SFA, saturated fatty acid; MUFA, monounsaturated fatty acid; PUFA, polyunsaturated fatty acid. All values are expressed as the mean ± SEM (n = 4). * and ** indicate significant differences (*p* < 0.05 and *p* < 0.01) between the NC and 0.8 g/kg Pro groups; ns indicates no significant difference.

## Data Availability

The raw data of 16S rRNA and transcriptome sequencing used in this study have been submitted to open database NCBI Sequence Read Archive (SRA) and the accession numbers were PRJNA1012120 and PRJNA1012106, respectively. All other data are contained within the main manuscript and supplementary material.

## Supplementary Materials

The following supporting information can be downloaded at: https://www.mdpi.com/article/10.3390/antiox12122095/s1, Figure S1: Schematic diagrams for experimental design and sampling; Figure S2: PCA score plot assessing quality of metabolomics data; Figure S3: Metabolome analysis of muscle between NC and Pro groups; Figure S4: DEGs in intestines of *C. carpio* between the NC and Pro groups; Figure S5: GO enrichment analysis for the DEGs in the intestines of *C. carpio* between the NC and Pro groups; Figure S6: KEGG enrichment analysis for the DEGs in the intestines of *C. carpio* between the NC and Pro groups; Figure S7: Venn diagram in ASVs (A), phylum (B), and genus (C) of intestinal microbiota in NC and Pro groups; Table S1: Formation and nutrient of the experimental diets [89]; Table S2: Specific primer sequences for qPCR in the study; Table S3: The hydrolyzed amino acid composition in muscle of C. carpio fed on normal diet (NC) and Pro-supplemented diet (Pro); Table S4: Valid data used in transcriptome analysis.
