# Supplementary material for: Multi-Omics Analysis to Understand the Effects of Dietary Proanthocyanidins on Antioxidant Capacity, Muscle Nutrients, Lipid Metabolism, and Intestinal Microbiota in Cyprinus carpio
Source: Antioxidants (Basel). 2023 Dec 10;12(12):2095. doi: 10.3390/antiox12122095 (PMC10740959; doi:10.3390/antiox12122095)
Supplement: Supplementary file 1 [file antioxidants-12-02095-s001.zip › antioxidants-2748073-supplementary.pdf]

Table S1 Formation and nutrient of the experimental diets [88]

Ingredients (g/kg)	Dietary Pro levels (g/kg)			
	0	0.2	0.4	0.8
Fish meal	100	100	100	100
Wheat middling	230	230	230	230
Rice bran	74	74	74	74
Soybean meal	140	140	140	140
Cotton seed meal	140	140	140	140
Rape seed meal	194	194	194	194
Corn germ meal	50	50	50	50
Vitamin premix [1]	10	10	10	10
Mineral premix [2]	10	10	10	10
Soybean oil	25	25	25	25
$Ca(H_2PO_4)$	10	10	10	10
Carboxymethylcellulose sodium	10.0	9.8	9.6	9.2
Choline chloride	1	1	1	1
Pro	0	0.2	0.4	0.8
Sodium chloride	6	6	6	6
Total	1000	1000	1000	1000
Composition				
Crude protein (%)	32.3	32.3	32.3	32.3
Crude lipid (%)	5.3	5.3	5.3	5.3
Ash (%)	5.4	5.4	5.4	5.4

Note:[1]Vitamins supplied per kg of diet:5000 IU; V_{B1} 10 mg; V_{D3} 1000 IU; V_{B2} 10 mg; V_{B12} 0.02 mg; V_E 50 mg; V_{B6} 4 mg; V_{K3} 2 mg; pantothenic acid 20 mg; folic acid 1 mg; niacin 20 mg; inositol 100 mg; biotin 0.2 mg; V_C 200 mg; [2]Minerals supplied per kg of diet: Fe 100 mg; Zn 80 mg; Mn 8 mg; Mg 100 mg; Cu 3 mg;I 0.3 mg; Co 0.05 mg; Se 0.1 mg.

Table S2. Specific primer sequences for qPCR in the study.

Gene	Primer sequence(5'-3')
sterol 26-hydroxylase, mitochondrial (*CYP27A1*)	F: GATCGCATCTGTGTGTCCGAATA<br>R: GCGTAGTGACAGAGATGGAACT
phosphoenolpyruvate carboxykinase 1 (*pck1*)	F: TGACCAGAGGAGGATCGTGT<br>R: TGCCCAGAATCAGCATGTGT
acyl-CoA synthetase long chain family member 1a (*acsl*)	F: GCCTGGATGGGCAAAGAAAAG<br>R: CTGGTCAGTCTGCTGTTTGAAG
CD36 molecule (*CD36*)	F: CCTCTCTCTACCCCACTTCCT<br>R: CCAGAACTGCCGTCTCGTTTA
peroxisome proliferator-activated receptor alpha (*pparα*)	F: AGTGACCTGATGGAGCCGAA<br>R: AAGAGTAAGGCGTTGTCGGG
carnitine palmitoyltransferase 1 (*cpt1*)	F: TTGACCTACAGTTGAGCCGC<br>R: ACACCCGTGAGAACACCATT
aquaporin 7 (*aqp7*),	F: TTGGGGCATGGAGGTTTTCA<br>R: ACTCCACCAATAAAGGGGGC
cathepsin A (*ctsa*)	F: CCGTGTGCTGGTGTATAACG<br>R: GAACATCGTGAAGGCCGCTA
galactosylceramidase a (*galca*)	F: GGTGGAGATGGGCTGATGAC<br>R: TGAGGTTCCCTTTACGGTCC
glucosidase, beta, acid (*gba*)	F: TACCGTTTCGTGAGAGTGCC<br>R: CACTCCAAGCACTGGCAAAC
N-acylsphingosine amidohydrolase 1b (*asah1b*)	F: CTTTTGTGTTTCTGTGCTTGTCTG<br>R: CCAGGACACATCCCCTTCAAA
acid phosphatase 5b, tartrate resistant (*acp5b*)	F: ATCAGCCCAGATCGCCTACT<br>R: TCCATATCAGATGTTGTTTGGCAG
phospholipase A2, group XV (*pla2g15*),	F: ATGGAGACGGAACGGTCAAC<br>R: GTTCAGCAGCATGGCAACAT
β-actin	F: ATCCGTAAAGACCTGTATGCCA<br>R: GGGGAGCAATGATCTTGATCTTCA

Table S3. The hydrolyzed amino acid composition in muscle of *C. carpio* fed on normal diet (NC) and Pro-supplemented diet (Pro)

Amino acids (g/100g, ww)	Groups		
	NC	Pro	Sig.
ASP	1.45 ± 0.04	1.29 ± 0.02	ns
Thr	0.66 ± 0.02	0.59 ± 0.01	ns
Ser	0.49 ± 0.01	0.45 ± 0.01	ns
Glu	1.94 ± 0.05	1.71 ± 0.03	ns
Gly	0.64 ± 0.02	0.6 ± 0.01	ns
Ala	0.93 ± 0.03	0.83 ± 0.02	ns
Cys	0.15 ± 0.01	0.13 ± 0.01	ns
Val	0.75 ± 0.03	0.65 ± 0.02	ns
Met	0.4 ± 0.02	0.35 ± 0.01	ns
Ile	0.68 ± 0.03	0.59 ± 0.02	ns
Leu	1.22 ± 0.05	1.08 ± 0.02	ns
Tyr	0.49 ± 0.02	0.44 ± 0.01	ns
Phe	0.66 ± 0.02	0.58 ± 0.01	ns
Lys	1.51 ± 0.05	1.33 ± 0.02	ns
His	0.53 ± 0.02	0.48 ± 0.01	ns
Arg	0.88 ± 0.03	0.78 ± 0.02	ns
Pro	0.47 ± 0.01	0.44 ± 0.01	ns
ΣEAA	7.28 ± 0.27	6.43 ± 0.14	ns
ΣNEAA	6.55 ± 0.2	5.87 ± 0.08	ns
ΣTAA	13.82 ± 0.46	12.29 ± 0.22	ns

EAA, essential amino acid; NEAA, non-essential amino acid; TAA, total amino acid; ww, wet weight; ns, no significant. The results are expressed as means ± SEM (n = 4).

Table S4. Valid data used in transcriptome analysis

Sample	Raw Data(bp)	Clean Data(bp)	Q_{20} (%)	Q_{30} (%)	GC (%)	Total mapped (%)
NC1	7,277,567,100	6,820,547,754	97.93	94.18	48.17	89.92
NC2	6,301,162,500	5,922,190,150	97.97	94.24	46.93	89.78
NC3	7,014,687,300	6,538,620,700	98.05	94.36	46.71	89.99
NC4	7,172,630,100	6,741,544,805	97.90	94.05	46.84	89.28
Pro1	7,467,783,300	7,005,632,038	98.13	94.63	47.01	88.97
Pro2	6,406,988,100	6,010,963,360	97.88	94.06	46.02	89.69
Pro3	5,730,362,700	5,392,441,669	97.93	94.14	45.55	89.24
Pro4	7,252,806,900	6,828,604,916	97.69	93.65	45.04	89.92

GC, GC content in clean reads, Q_{20} and Q_{30} the base quality score (Q score) was no less than 20 and 30, respectively, in clean reads. NC, normal control group, Pro, Pro treatment group.

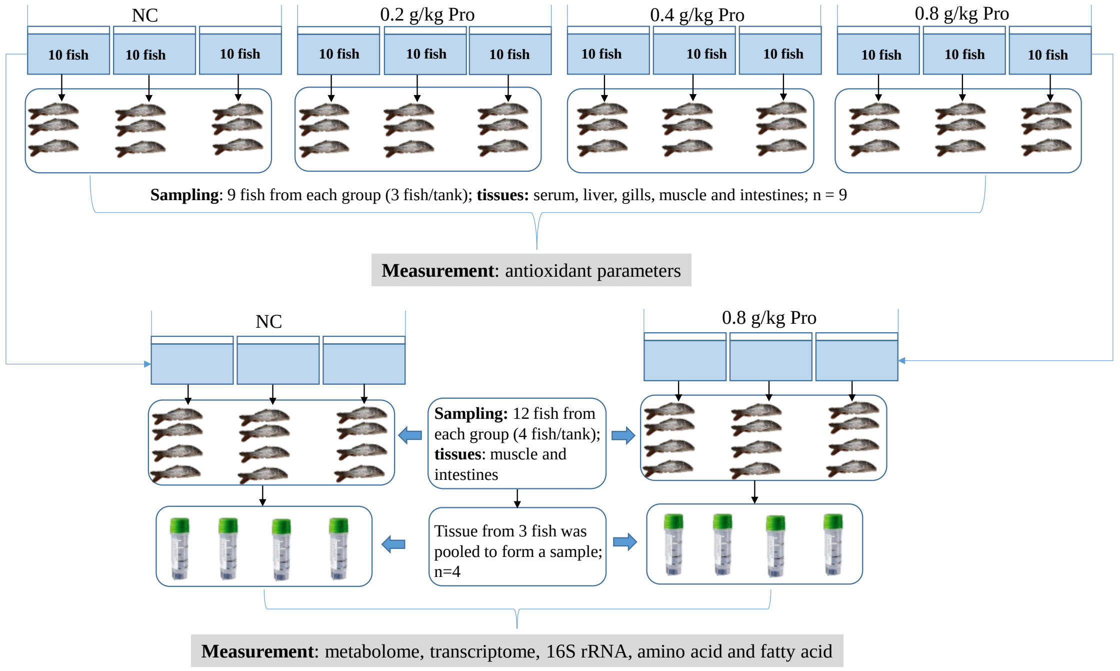

Figure S1. Schematic diagrams for experimental design and sampling

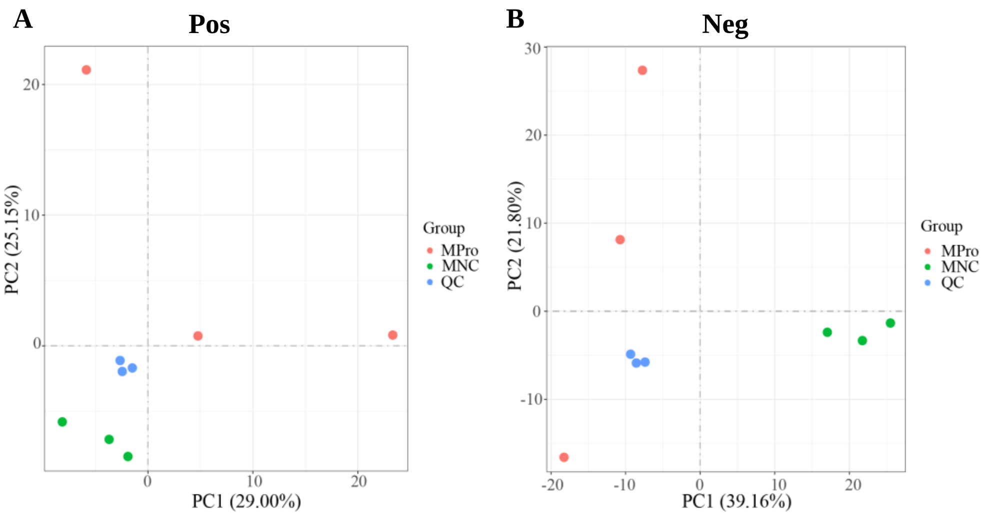

Figure S2. PCA score plot assessing quality of metabolomics data. (**A,B**) Metabolite profiles identified in positive (Pos) and negative (Neg) ion modes, respectively. MNC, NC group; MPro, Pro-fed group; QC, quality control samples. The QC samples exhibit tight clustering in both plots, indicating that the detection method is stable and the data is reliable.

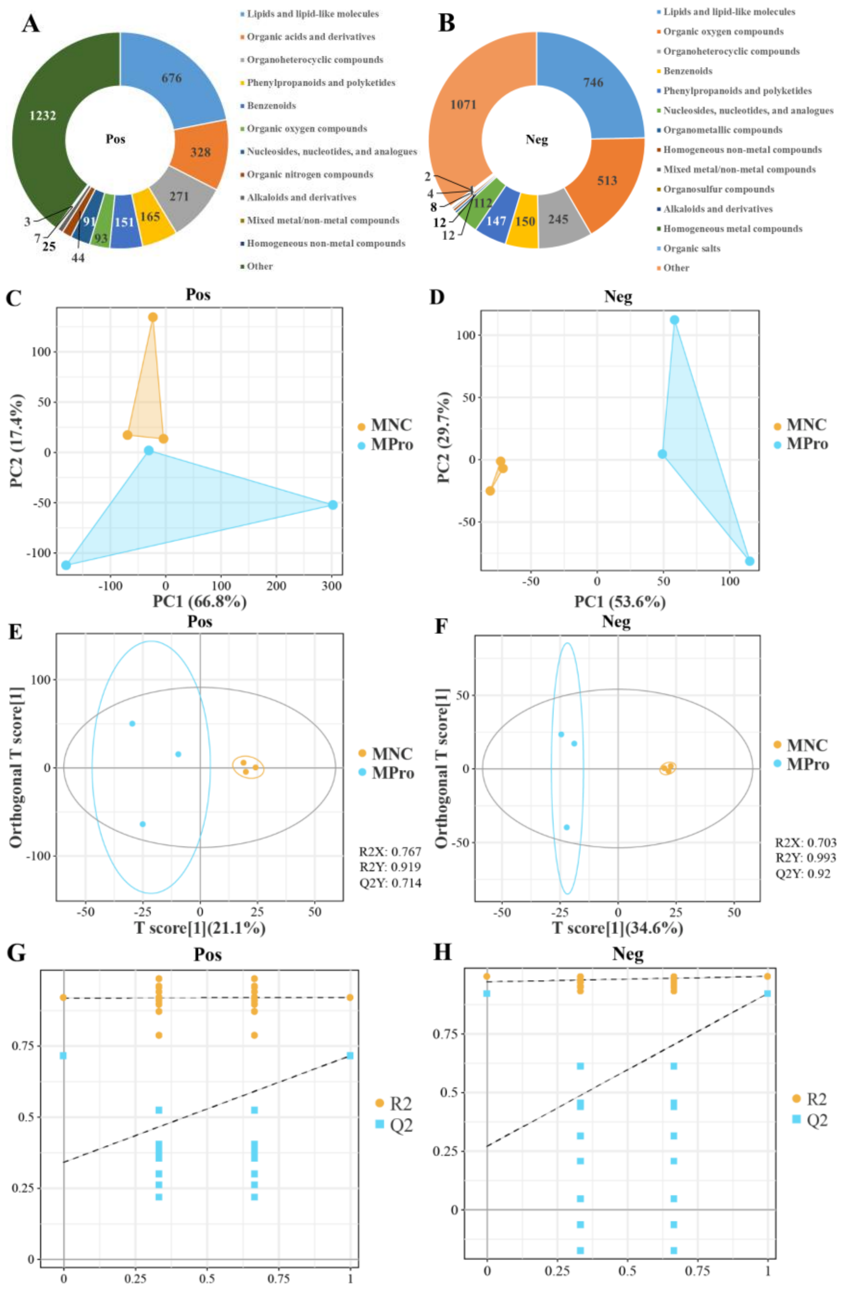

Figure S3. Metabolome analysis of muscle between NC and Pro groups. (**A,B**) Numbers and classification of the annotated metabolites in positive (Pos) and negative (Neg) ion modes. (**C,D**) PCA score plot of samples in MNC and MPro under Pos and Neg ion modes. (**E,F**) OPLS-DA score plot of samples in MNC and MPro under Pos and negative Neg ion modes. (**G,H**) Response-sorting test of the OPLS-DA in Pos and Neg ion modes.

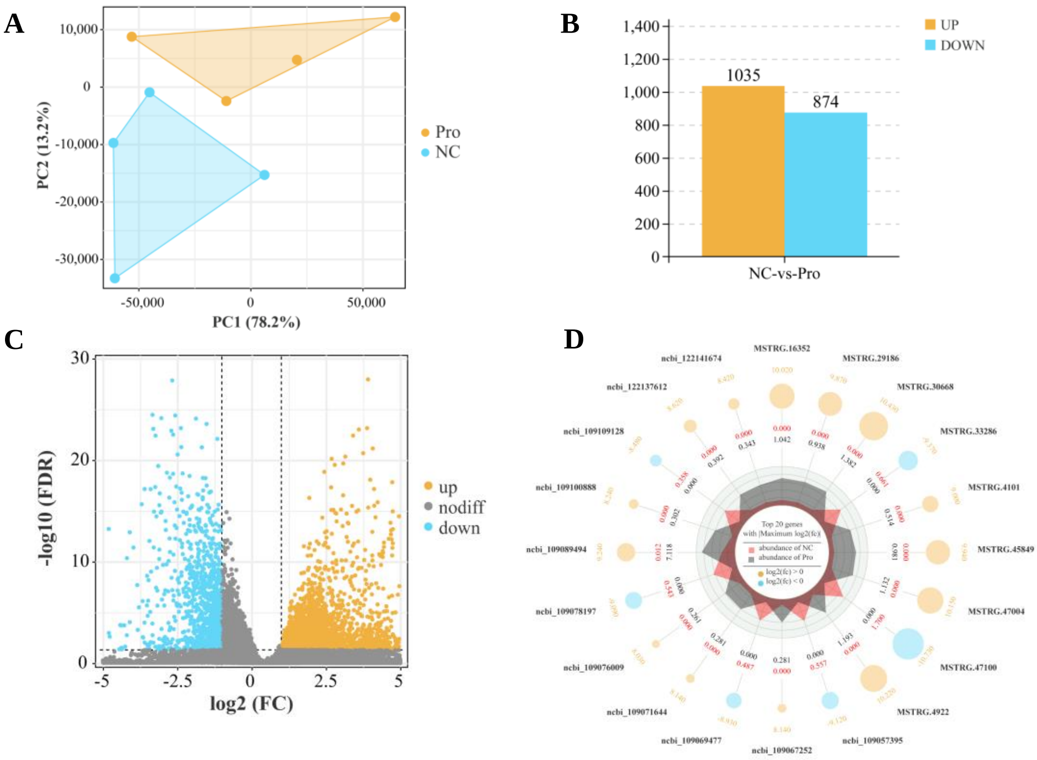

Figure S4. DEGs in intestines of *C. carpio* between the NC and Pro groups. (**A**) Correlation among samples in the NC and Pro groups. (**B**) Significantly up-regulated and down-regulated genes number in different groups. (**C**) Volcano plot of identified genes including up-regulated and down-regulated genes in the RNA-seq. (**D**) Top 20 DEGs induced by Pro treatment.

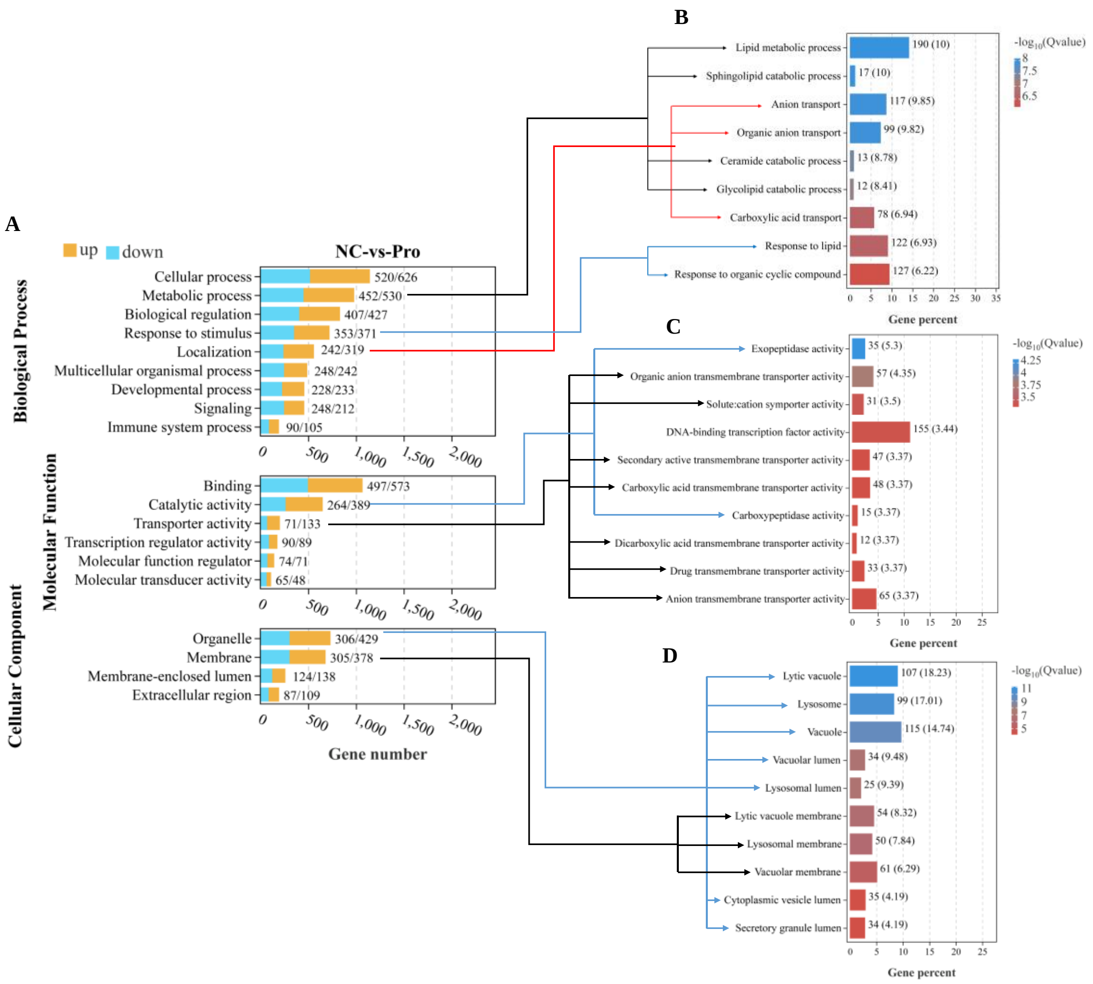

Figure S5. GO enrichment analysis for the DEGs in the intestines of *C. carpio* between the NC and Pro groups. (**A**) GO enrichment terms of DEGs in level 1 GO term and level 2 GO term. (**B**) The top 10 GO terms in the biological process category. (**C**) The top 10 GO terms in the molecular function category. (**D**) The top 10 GO terms in the cellular component category.

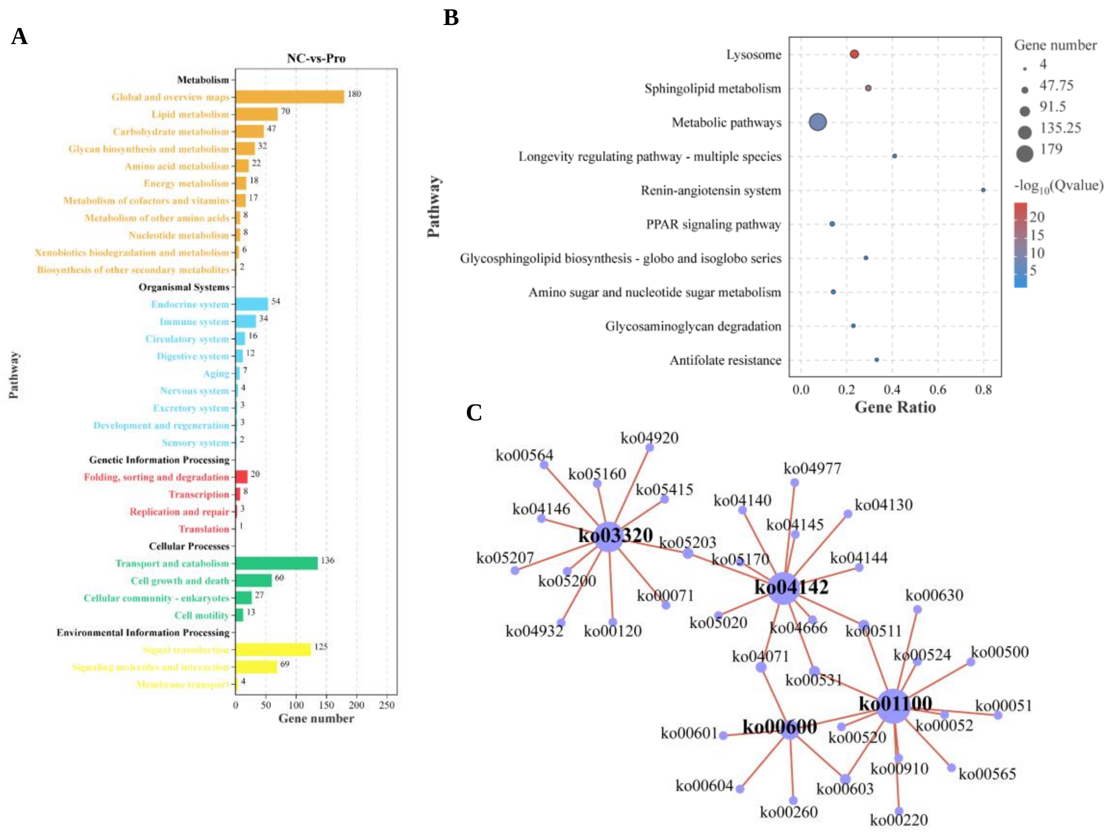

Figure S6. KEGG enrichment analysis for the DEGs in the intestines of *C. carpio* between the NC and Pro groups. (**A**) DEGs enrichment in the KEGG A Class and B Class. (**B**) The top 10 enriched KEGG pathways. (**C**) Network plot of the interactions between different pathways after Pro feeding.

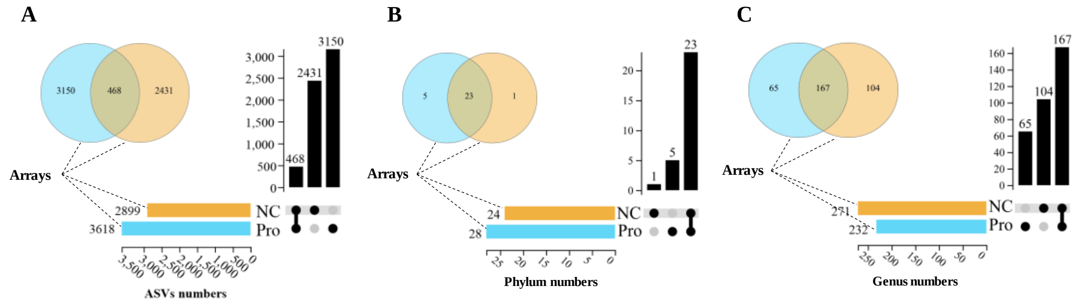

Figure S7. Venn diagram in ASVs (**A**), phylum (**B**), and genus (**C**) of intestinal microbiota in NC and Pro groups.
